# Effects of transcranial electrical stimulation on face identification and related perceptual processes: a systematic review

**DOI:** 10.1007/s00426-026-02313-6

**Published:** 2026-06-25

**Authors:** Aline Miranda de Vasconcelos, José Aldecyano Lino Gomes, Paulo Frassinetti Delfino do Nascimento, Géssika Araújo de Melo, Laurent Koessler, Nelson Torro-Alves

**Affiliations:** 1 Cognitive Neuroscience and Behavior Program, Federal University of Paraíba, Cidade Universitária, João Pessoa, Paraíba 58051-900 Brazil; 2 State University of Paraíba, Campina Grande, Brazil; 3 Université de Lorraine, CNRS, IMoPA, Nancy, France; 4 National Institute of Science and Technology on Social and Affective Neuroscience, São Paulo, Brazil

**Keywords:** Transcranial electrical stimulation, Non-invasive brain stimulation, Face identification, Face recognition

## Abstract

Face identification is a core capacity underpinning effective social interaction and communication. Although a growing body of literature has examined the effects of transcranial electrical stimulation (TES) on face identification, findings remain fragmented and lack an integrative synthesis. This systematic review adopted a broad scope, encompassing all published studies on TES and face identification, with the aim of mapping methodological trends and substantive findings, rather than focusing on a specific subtype of intervention or population. We conducted a PRISMA-guided systematic review of TES effects on face identification. From an initial pool of 261 records, 28 studies met the eligibility criteria. Overall, the evidence highlights both the potential and the complexity of using TES to modulate face identification processing. Targeted stimulation of occipital and fusiform regions shows promising effects in some paradigms; however, inconsistent results and notable methodological limitations preclude definitive conclusions. In particular, small, demographically homogeneous samples limit generalizability, while heterogeneity in stimulation parameters, task designs, and outcome measures complicate cross-study comparisons. By underscoring the need for rigorously controlled experiments with greater anatomical precision, especially in clinical populations, this review aims to inform the development of more robust, transparent methodological guidelines and to support future investigations with stronger scientific relevance.

## Introduction

Face identification is an essential skill for social interaction and effective communication between individuals (Souza et al., 2008). Although face identification abilities begin to develop very early, shaped by multisensory inputs (Leleu et al., 2020; Rekow et al., 2024), this expertise persists throughout life, as most individuals become experts at discriminating faces and can recognize more than 5,000 faces (Jenkins et al., 2018). In this review, we adopt the term “face identification” in a broad sense, encompassing both identity-related processing and associated perceptual processes (e.g., discrimination, matching, and orientation judgments), regardless of whether they involve explicit memory demands.

Two main approaches to visual processing have been proposed to explain the mechanisms underlying face identification. The first, the analytical approach, holds that observers initially attend to relevant facial features, processing them separately before integrating them into a coherent representation (Davies et al., 1977; Van Belle et al., 2011). In contrast, the holistic approach posits that faces are perceived as unified wholes, with recognition driven by the configuration of features and their spatial relations, which enables rapid and efficient identification (Rossion, 2008). This approach is supported by phenomena like the whole-face effect, where individuals are typically more adept at recognizing faces when they are shown as complete entities rather than in separate parts (Tanaka & Farah, 1993). Additionally, the composite-face effect illustrates how facial features are effortlessly combined into a unified perception (Rossion, 2013; Young et al., 1987). Importantly, these processes are not mutually exclusive; the extent to which individuals rely on analytical or holistic processing may vary according to task demands and individual differences in face identification ability (Leong et al., 2023).

Face identification involves distinguishing structural features that remain constant, despite variations in facial expressions and movements of the eyes and mouth (Bruce & Young, 1986). Therefore, face perception requires processing not only the invariant aspects, such as facial identity, but also the variant aspects, like facial emotions (Corrow et al., 2016). Studies have consistently supported the dissociation between facial identity processing and facial emotion recognition (Bruce & Young, 1986). A notable example of this is observed in individuals with prosopagnosia, a condition characterized by difficulty in identifying faces, while the ability to recognize facial emotions typically remains intact (Bentin et al., 2007; Etcoff, 1984; Rossion, 2022). Conversely, in certain psychiatric conditions, such as schizophrenia (Comparelli et al., 2013), bipolar disorder (Surguladze et al., 2005), and autism spectrum disorder (Baron-Cohen et al., 1999), individuals often exhibit more pronounced impairments in the recognition of facial emotions. This distinction highlights the independent nature of the cognitive processes underlying facial identity and emotion recognition. Although these findings support a functional distinction between identity and emotion processing, it is crucial to recognize their interaction (Gobbo et al., 2024). Emotional expressions can modulate identity recognition, with neural structures such as the superior temporal sulcus integrating dynamic and changeable cues with invariant facial features (Gobbo et al., 2024; Haxby et al., 2000).

Electrophysiological findings have significantly advanced the understanding of face perception. Scalp electroencephalography (EEG) studies have consistently identified the N170 component, a negative deflection occurring approximately 170 milliseconds after stimulus onset, as a reliable marker of early face-specific processing (Jacques et al., 2019). The N170 is typically maximal over lateral occipitotemporal electrodes and is more prominent for faces than other object categories, reflecting the structural encoding of facial features. Intracerebral recordings further support the existence of a specialized face processing network (Rossion et al., 2000), revealing selective and temporally precise face-related responses in areas such as the fusiform gyrus, inferior occipital cortex, and superior temporal sulcus. Electrical stimulation of these face-selective regions can induce distortions in face perception or even facial hallucinations (Jonas et al., 2014, 2015), providing causal evidence of their involvement. A comprehensive mapping of face-selective cortical regions has delineated a hierarchically organized face perception network (Jonas et al., 2016), offering a solid electrophysiological foundation for research into how non-invasive brain stimulation techniques, such as transcranial electrical stimulation (TES), might modulate face processing mechanisms.

Importantly, among the studies that have combined tDCS with EEG to investigate face processing, differences in the timing of stimulation relative to recording deserve attention. While some studies have employed a sequential approach, in which tDCS is administered either before or after EEG recording to avoid stimulation-induced artifacts (e.g., Yang et al., 2014), others have adopted a true concurrent tDCS-EEG co-registration protocol. For instance, Civile and colleagues (Civile et al., 2020, 2024) successfully implemented simultaneous tDCS-EEG using compatible systems (e.g., Starstim, Neuroelectrics), allowing for online monitoring of brain activity during active stimulation. This methodological distinction is critical, as simultaneous recording enables the direct assessment of tDCS-induced changes in neural responses (e.g., N170 latency and amplitude) while stimulation is ongoing, offering a more precise window into the online modulatory effects of tDCS on face-selective processing. In contrast, sequential designs may capture after-effects of stimulation but cannot rule out state-dependent changes that occur only during current delivery.

Complementing these electrophysiological insights, neuroimaging studies have identified the network responsible for face perception, which primarily consists of three regions within the occipitotemporal visual cortex: the inferior occipital gyri, the fusiform gyrus, and the superior temporal sulcus. These regions play pivotal roles in face processing, contributing to various aspects of facial perception (Hoffman & Haxby, 2000). The Occipital Face Area (OFA), located in the inferior occipital gyrus of the occipital lobe, plays a crucial role in the early stages of face perception. The OFA is primarily involved in the structural encoding of facial features, such as the eyes, nose, and mouth, and is responsible for processing these individual components rather than the holistic face. The fusiform gyrus stands out as the principal region associated with the processing of facial identity (Grill-Spector et al., 2006). Meanwhile, the superior temporal sulcus is engaged in processing dynamic facial cues, such as gaze direction, lip movement, and expressions of emotion (Haxby et al., 2000).

In addition to these core regions, the processing of facial emotions involves an extended network of brain areas. These include the amygdala, basal ganglia, lateral and medial parietal cortex, medial temporal lobe, lateral temporal cortex, dorsal and rostral anterior cingulate cortex, anterior insular cortex, and prefrontal cortex (Lieberman, 2007). Further specialization exists within this extended network: for instance, the intraparietal sulcus contributes to spatial attention, and the anterior temporal pole supports access to semantic information and identification of personally familiar individuals, such as names (Hoffman & Haxby, 2000; Sellal, 2022).

The demonstration that direct electrical stimulation of face-selective regions causally disrupts face perception (Jonas et al., 2014, 2015) raises the question of whether non-invasive neuromodulation techniques could similarly influence face identification processes. In this context, non-invasive brain stimulation techniques have emerged as valuable tools to modulate brain activity, offering both therapeutic potential for psychopathological conditions (de Melo et al., 2020; Lyra de Brito Aranha et al., 2024) and the ability to investigate the neural correlates of cognitive functions and their functional connectivity (Nejati et al., 2021).

Transcranial Direct Current Stimulation (tDCS) is a widely used non-invasive brain stimulation technique, often studied for its potential in modulating cortical excitability and neural activity. This technique delivers low-intensity continuous electrical currents, generally ranging from 0.5 to 4.0 mA (Bikson et al., 2009). However, in the context of facial identification studies, currents between 1.0 mA and 2.0 mA are often used, with stimulation sessions typically lasting from 10 to 30 min (Nejati et al., 2021). The electrical currents are administered through two electrodes, usually sized between 25 cm² and 35 cm², which are placed in direct contact with the scalp. At the neurophysiological level, anodal stimulation generally promotes neuronal depolarization and increased excitability, whereas cathodal stimulation is associated with hyperpolarization and reduced neuronal firing (Thair et al., 2017). However, the translation of these neurophysiological effects into behavioral outcomes is more complex. While anodal stimulation is often associated with facilitation of tasks engaging the targeted region and cathodal stimulation with inhibition, these behavioral effects are not absolute and can vary considerably depending on stimulation parameters (e.g., intensity, duration, electrode montage), individual differences, and the cognitive state of the participant during stimulation (Nitsche et al., 2008; Tremblay et al., 2014). In some cases, polarity-dependent effects may be reversed or absent, highlighting the importance of considering these moderating factors when interpreting tDCS findings (Li et al., 2015; Opitz et al., 2015).

Besides tDCS, other forms of transcranial electrical stimulation have been employed to investigate face perception, including transcranial random noise stimulation (tRNS) and transcranial alternating current stimulation (tACS). tRNS delivers alternating current at randomly varying frequencies within a broad spectrum (typically 101–640 Hz), which is thought to increase cortical excitability by inducing stochastic resonance effects and modulating sodium channel dynamics (Terney et al., 2008; Krause & Cohen Kadosh, 2013). Unlike tDCS, tRNS does not exhibit polarity-dependent effects and has been shown to enhance performance in various perceptual and cognitive domains, including working memory and perceptual learning (Reed & Cohen Kadosh, 2018; Murphy et al., 2020). In the domain of face identification, findings remain inconclusive. One study reported that high-frequency tRNS over occipito-temporal sites (P7/P8) improved performance on the Cambridge Face Perception Test, an effect that was face-specific as it did not generalize to inverted faces (Romanska et al., 2015). However, subsequent studies have reported mixed results, with some showing no effects or even inhibitory effects depending on stimulation parameters, electrode montage, and experimental design (Penton et al., 2018; Willis et al., 2019). These discrepant findings highlight the need for further research to clarify the conditions under which tRNS modulates face identification.

In contrast, tACS delivers a sinusoidal alternating current at a specific frequency, allowing for the entrainment of endogenous neural oscillations (Cabral-Calderin & Wilke, 2020; Polania et al., 2018). By synchronizing or desynchronizing oscillatory activity in targeted brain regions, tACS can modulate frequency-specific neural processes associated with cognitive functions such as attention, memory, and perception (Antal & Herrmann, 2016; Herrmann et al., 2013). Unlike tDCS, which exerts its effects through tonic shifts in cortical excitability, tACS operates by interacting with ongoing oscillatory dynamics, offering a more targeted approach to modulating the timing of neural activity. Both tRNS and tACS offer complementary approaches to tDCS, expanding the methodological toolkit for investigating the neural basis of face identification.

Moreover, TES protocols commonly incorporate a placebo (sham) condition, where a simulated current is briefly activated for a short period (e.g., 15–30 s) before being automatically turned off, allowing for controlled experimental conditions. This integration of technical precision and methodological flexibility makes these techniques valuable tools in both research and clinical settings, particularly in exploring and modulating face identification and related perceptual processes and their underlying neural substrates (DaSilva et al., 2011; Terney et al., 2008).

To date, most systematic reviews have concentrated on the effects of TES in cognitive domains such as attention, memory, and executive functions. For tDCS, several reviews have summarized its effects in both healthy individuals (Dedoncker et al., 2016; Katsoulaki et al., 2017) and in clinical groups (Khan et al., 2022). For tRNS, systematic reviews have demonstrated its safety and efficacy in psychiatric disorders, including ADHD, depression, and schizophrenia (Tripathi et al., 2026; Yin et al., 2024). For tACS, meta-analyses have shown improvements in working memory, attention, and executive control across healthy, aging, and clinical populations (Grover et al., 2023; Lee et al., 2023). However, the application of TES techniques, including tDCS, tRNS, and tACS, in the domain of face identification remains underexplored. This research gap was underscored in a systematic review that specifically assessed the impact of neuromodulation on facial emotion recognition (Nejati et al., 2022), which, to our knowledge, represents the only comprehensive synthesis in the broader domain of face processing, leaving face identification largely unexamined.

Here, we conducted a systematic review of studies that evaluate the effects of TES (tDCS, tRNS, and tACS) on face identification. This review aimed to summarize current results, identify trends, and highlight gaps in research on the influence of these neuromodulation techniques on face identification. Although TES interventions have been applied in diverse contexts, including cognitive rehabilitation, emotional modulation, and facial perception studies, this review deliberately adopted a broad approach, encompassing all studies that investigated the effects of tDCS, tRNS, and tACS on face identification. We chose not to restrict the scope to a specific type of intervention (e.g., clinical vs. experimental studies) or a single population, as our primary aim was to map the methodological and substantive landscape of the existing literature, identifying trends, inconsistencies, and gaps that may guide future, more focused investigations. To that end, we compiled information on the main characteristics of study design (participants and type of cognitive task), stimulation parameters (type of stimulation, application area, intensity, duration, electrode size, online/offline timing, number of sessions), and the populations studied.

## Methods

The systematic review was conducted following the PRISMA guidelines (Page et al., 2021). The review protocol was registered with PROSPERO under the number CRD42024574902. The electronic search was conducted in March 2026 in the databases PubMed, Web of Science, Psycinfo, and Science Direct. The following descriptors were used: ((“transcranial electrical stimulation” OR “non-invasive brain stimulation” OR “tDCS” OR “tACS” OR “tRNS”) AND (“face memory” OR “face perception” OR “facial recognition” OR “facial identity recognition” OR “face recognition” OR “face identification”). These keywords were selected to maximize sensitivity, even in the absence of specific MeSH terms.

### Inclusion and exclusion criteria

The study selection was based on the PICOS framework:

- **Population (P)**: Healthy individuals or patients with neurological or psychiatric conditions affecting facial recognition.
- **Intervention (I)**: Studies utilizing tDCS, tACS or tRNS as the primary intervention to modulate face identification.
- **Control (C)**: Studies with a control group, either a placebo (sham) group or a non-intervention comparison group.
- **Outcome (O)**: Studies measuring the effects of tDCS, tACS or tRNS on face identification, based on behavioral and electrophysiological measures.
- **Study Type (S)**: Clinical trials and intervention studies examining the effects of tDCS, tACS or tRNS on face identification.

Only experimental studies with a randomized, sham-controlled design published in English were included. Exclusions were made for duplicates, animal studies, other stimulation forms, pharmacological interventions, case studies, reviews, reports, and studies not focused on face identification. No restrictions were placed on the year of publication.

### Study selection

Study selection was conducted by two reviewers (AM and GA) using the Rayyan platform, with blinding between the reviewers during their analysis. After excluding duplicate studies across databases, articles were selected by reviewing the title and abstract based on the eligibility criteria. If there was no consensus between the two reviewers, a third reviewer (NT) was asked to read the full text to determine the inclusion or exclusion of the study in the review.

### Data extraction and analysis

Two independent reviewers (AM and AG) extracted data from each study using an Excel spreadsheet. As mentioned before, the review considered both electrophysiological and behavioral outcomes.

### Risk of bias and data summary

The methodological quality of the studies was assessed using the PEDro scale (Beaton et al., 2000). The results are presented in Tables 1 and 2. Each study was classified as high, moderate, or low quality based on its total score. Studies scoring between 8 and 10 were considered high quality, as they provided more robust evidence. Those scoring between 4 and 7 were deemed to have moderate quality, while studies scoring below 4 were classified as low quality due to limitations that may have affected the validity of the results. It was observed that approximately 71% of the studies included in the review demonstrated moderate methodological quality, while 21% were classified as high quality and 8% as low quality.

**Table 1:** Methodological quality of included studies according to the PEDro scale (ordered by total score)

Author (s)	TOTAL (methodological quality score according to the PEDro scale)
Civile et al. (2020)	9
Alekseichuk et al. (2020)	8
Civile et al. (2025)	8
Civile and McLaren (2022)	8
Civile et al. (2021a) ^a^	8
Civile et al. (2021b) ^b^	8
Civile et al. (2018)	8
Awasthi (2022)	7
Estudillo et al. (2023)	7
Civile et al. (2024)	7
Civile et al. (2023)	7
Payne & Tasakiris (2017)	7
Civile et al. (2016)	7
Saghravanian and Esteky (2025)	6
Gonzalez-Perez et al. (2019)	6
Penton et al. (2018)	6
Romanska et al. (2015)	6
Kho et al. (2023b) ^b^	6
Willis et al. (2019)	6
Civile et al. (2019)	6
Barbieri et al. (2016)	6
Smirni et al. (2015)	6
Kho et al. (2023a) ^a^	5
Papagno et al. (2021)	5
Costantino et al. (2017)	5
Brunyé et al. (2017)	5
Renzi et al. (2015)	4
Yang et al. (2014)	4

**Table 2:** Evaluation of studies using the PEDro scale to analyze risk of bias

PEDro scale	Yang et al. (2014)	Renzi et al. (2015)	Smirni et al. (2015)	Romanska et al. (2015)	Barbieri et al. (2016)	Civile et al. (2016)	Brunyé et al. (2017)	Costantino et al. (2017)	Payne & Tasakiris (2017)
Eligibility criteria specified	YES	YES	YES	YES	YES	YES	YES	YES	YES
Random allocation	YES	NO	YES	YES	YES	YES	YES	NO	YES
Concealed allocation	NO	NO	NO	NO	NO	NO	NO	NO	NO
Groups similar at baseline	NO	NO	YES	YES	YES	YES	YES	YES	YES
Blinding of participants	YES	YES	YES	YES	YES	YES	NO	YES	YES
Blinding of therapists	NO	NO	NO	NO	NO	YES	NO	NO	NO
Blinding of assessor	NO	NO	NO	NO	NO	YES	NO	NO	YES
Follow-up > 85%	NO	YES	YES	YES	YES	NO	YES	YES	YES
Intention-to-treat analysis	NO	NO	NO	NO	NO	NO	NO	NO	NO
Between-group statistical comparisons	YES	YES	YES	YES	YES	YES	YES	YES	YES
Point measures and measures of variability for at least one key outcome	YES	YES	YES	YES	YES	YES	YES	YES	YES
Civile et al. (2018)	Penton et al. (2018)	Civile et al. (2019)	Gonzalez-Perez et al. (2019)	Willis et al. (2019)	Alekseichuk et al. (2020)	Civile et al. (2020)	Civile et al. (2021a) ^a^	Civile et al. (2021b) ^b^	Papagno et al. (2021)
YES	YES	YES	YES	YES	YES	YES	YES	YES	YES
YES	YES	YES	YES	YES	YES	YES	YES	YES	NO
NO	NO	NO	NO	NO	NO	YES	YES	YES	NO
YES	YES	YES	YES	YES	YES	YES	YES	YES	YES
YES	YES	YES	YES	YES	YES	YES	YES	YES	YES
YES	NO	NO	NO	NO	NO	YES	YES	YES	YES
YES	NO	NO	NO	NO	YES	YES	YES	YES	NO
YES	YES	YES	YES	YES	YES	YES	NO	NO	NO
NO	NO	NO	NO	NO	YES	NO	NO	NO	NO
YES	YES	YES	YES	YES	YES	YES	YES	YES	YES
YES	YES	YES	YES	YES	YES	YES	YES	YES	YES
Awasthi (2022)	Civile and McLaren (2022)	Kho et al. (2023a) ^a^	Civile et al. (2023)	Estudillo et al. (2023)	Kho et al. (2023b) ^b^	Civile et al. (2024)	Civile et al. (2025)	Saghravanian and Esteky (2025)	
YES	YES	YES	YES	YES	YES	YES	YES	YES	
YES	YES	YES	YES	YES	NO	YES	YES	YES	
NO	YES	NO	NO	NO	NO	NO	YES	NO	
YES	YES	YES	YES	YES	YES	YES	NO	NO	
YES	YES	YES	YES	YES	YES	YES	YES	YES	
NO	YES	NO	NO	NO	NO	YES	YES	NO	
NO	NO	NO	YES	YES	NO	YES	YES	YES	
YES	YES	NO	YES	YES	YES	NO	YES	YES	
YES	NO	NO	NO	NO	YES	NO	NO	NO	
YES	YES	YES	YES	YES	YES	YES	YES	YES	
YES	YES	YES	YES	YES	YES	YES	YES	YES	

The studies included in Table 3 (which would be a comprehensive summary table in the final manuscript) were summarized using the following variables: (1) identifying author, (2) participants, (3) type of task, (4) electrophysiological measurements, (5) Non-invasive Brain Stimulation Parameters (such as application area, intensity, duration, electrode size, and type of tDCS), and (6) main results.

**Table 3:** Characteristics of the studies included in the review that evaluated the effects of the tDCS on face recognition

Author (s)	Participants:	Type of tasks:	Electrophysiological measurements	Non-invasive Brain Stimulation Parameters	Main results
Yang et al. (2014)	Experiment 1: 24 (11 women), mean age of 22.8 years; Experiment 2: 39 (21 women), mean age of 22.7 years;	*Face Orientation Judgment Task*, *Composite Face Task* Behavioral measurements: Accuracy, Response Time and Sensitivity.	EEG: ERP components analyzed: *P1*,* N170*,* N250*	Application area: P7/P8; Intensity: 1.5 mA; Duration: 15 min. Electrode size: 35 cm²; Stimulation type: cathodal and anodal. Type of tDCS: offline e online. Sessions = 3 (crossover; ≥ 3 days interval).	tDCS significantly reduced N170 amplitude in the right hemisphere (LaRc vs. sham: *p* =.015; LcRa vs. sham: *p* =.006). Additionally, the composite face effect was reduced (LaRc vs. sham: *p* =.011; LcRa vs. sham: *p* =.003), indicating decreased holistic face processing. No effects were observed on N170 latency or in the left hemisphere.
Romanska et al. (2015)	Experiment 1: 36 (23 women), mean age of 27 years. Experiment 2: 40 (22 women), mean age of 27.17 years	Cambridge Face Perception Test–Identity (CFPT-Identity) Cambridge Face Perception Test–Trustworthiness (CFPT-Trustworthiness). Behavioral measurements: Accuracy/percentage correct	No.	Application area: P7/P8 (Experiment 1); P7/P8 or C3/C4 (Experiment 2); Intensity: 1 mA; Duration: 20 min. Electrode size: 25 cm²; Stimulation type: active high-frequency. tRNS and sham (Experiment 1), active high-frequency occipitotemporal tRNS and sensorimotor tRNS (Experiment 2). Type of non-invasive brain stimulation: high-frequency tRNS. Type of stimulation: offline. Sessions = 1 (between-subjects, single-session design, no interval).	Experiment 1: active tRNS over lateral occipitotemporal cortices selectively improved performance on upright CFPT-Identity relative to sham, with a significant Task Type × Group interaction, F (2,68) = 3.34, *p* =.041, η²*p* =.089; pairwise comparison for upright trials, *p* <.01. No effects were found for inverted CFPT-Identity trials (*p* =.368) or CFPT-Trustworthiness (*p* =.841). Experiment 2: tRNS over P7/P8 produced better performance than sensorimotor stimulation, with a significant main effect of group, F (1,38) = 17.17, *p* <.001, η²*p* =.311. This effect was mainly driven by upright face trials, t (38) = 5.38, *p* <.001, with no significant difference for inverted trials, t (38) = 1.71, *p* =.095.
Renzi et al. (2015)	Experiment 1: 16 (13 women), mean age of 23.31 years; Experiment 2: 16 (11 women), mean age of 24.87 years;	*Mooney Faces Task*, *Composite Faces Task* Behavioral measurements: Accuracy, RTs, and Detection Sensitivity.	No.	Application area: OFA; Intensity: 2 mA; Duration: 20 min. Electrode size: 35 cm²; Stimulation type: anodal. tDCS type: offline. Sessions = 2 (crossover, consecutive days).	Anodal tDCS over the OFA impaired performance in both face and object detection tasks compared to sham (*p* <.05), suggesting a causal role in visual detection under degraded conditions. However, no significant effect was found on holistic face processing in the composite face task (*p* >.07), indicating the OFA may not be directly involved in holistic discrimination of facial identity.
Smirni et al. (2015)	36 (32 women). Experiment 1: 20, mean age of 23,56 years. Experiment 2: 16, mean age of 24,7 years.	*Recognition Memory Test (Italian version)*, *Faces Recognition Test* Behavioral measurements: Accuracy, RTs.	No.	Application area: F3/F4; Intensity: 1 mA; Duration: 20 min. Electrode size: 35 cm²; Stimulation type: cathodal and anodal. tDCS type: offline. Session = 2 (crossover, interval ≈ 6 h).	Cathodal tDCS over the right DLPFC significantly improved nonverbal recognition memory accuracy. Specifically, participants showed a statistically significant improvement compared to sham tDCS (F = 7.84; *p* <.01). Additionally, the effects of right cathodal tDCS significantly differed from those of left cathodal tDCS (F = 14.54; *p* <.01). However, there was no significant modulation of reaction times.
Barbieri et al. (2016)	48 (33 women), mean age of 27 years;	*CFMT*, Cambridge Car Memory Test (*CCMT)*,* FP and OP* Behavioral measurements: Accuracy, RTs.	No.	Application area: PO8; Intensity: 1,5 mA; Duration: ETCC Online: 24.6 min and ETCC Offline: 20. Electrode size: 25 cm²; Stimulation type: anodal. tDCS type: online e offline. Sessions = 1 (between-subjects, no crossover)	The improvement in accuracy for offline tDCS compared to sham and online tDCS was statistically significant (*p* =.017 and *p* =.016, respectively). This indicates that offline tDCS enhanced performance by approximately 7% compared to the other conditions.
Civile et al. (2016)	Experiment 1: 32 (23 women), mean age of 20 years; Experiment 2: 72 (54 women), mean age of 22 years;	*Old/New Recognition Task* Behavioral measurements: Accuracy.	No.	Application area: Fp3; Intensity: 1.5 mA; Duration: 10 min. Electrode size: 35 cm²; Stimulation type: Experiment 1: anodal and Experiment 2: anodal and cathodal. tDCS type: online. Sessions = 1 (between-subjects, no crossover)	In Experiment 1, the Familiarity × Orientation interaction was significant in the Sham condition (F (1, 15) = 12.59, *p* =.001), but not in the Anodal condition. In Experiment 2, the three-way Familiarity × Orientation × Stimulation interaction was significant (F (1, 70) = 4.28, *p* =.042), showing that anodal stimulation had a distinct effect compared to cathodal stimulation. This result supports the view that anodal tDCS at Fp3 directly influences this modulation process to eliminate and possibly reverse perceptual learning.
Brunyé et al. (2017)	Active group: 24 (15 women), mean age of 21.3 years; Control group: 24 (17 women), mean age of 19.6 years;	*CFMT and Working Memory Task* Behavioral measurements: Cowan’s K, Hit Rate, False Alarm Rate, Response Time and Response Criterion.	No	Application area: PO10; Intensity: 0.5 mA and 1.5 mA; Duration: 10 min. Electrode size: Not specified. Note: In this study, two sintered Ag/AgCl ring electrodes were used in a 74-channel EasyCap (EEG cap) with plastic holders; Stimulation type: anodal. tDCS type: online. Session = 2 (crossover, interval ≥ 24 h).	tDCS targeting the right fusiform gyrus (1.5 mA) significantly improved working memory for faces at high memory loads. Post hoc tests showed improvements at load 4 (*p* =.004) and load 3 (*p* =.01), with no impact on non-face objects. Individuals with lower baseline performance showed greater benefits (*r* = −.54, *p* =.007).
Costantino et al. (2017)	86 (45 women), mean age of 26.65 years;	Cambridge Face Perception Test *(CFPT)*,* FP*,* OP*,* CFMT and CCMT* Behavioral measurements Accuracy, RTs.	No.	Application area: PO8; Intensity: 1.5 mA; Duration: 20 min. Electrode size: 25 cm²; Stimulation type: cathodal. tDCS type: offline. Sessions = 2 (mixed design, pre-post/offline stimulation design with between-subjects sham vs. cathodal groups and within-subjects task factor, same-day interval).	No overall effect of cathodal tDCS on performance was found (F (1,84) = 0.94, *p* =.34, η²*p* =.011). However, exploratory analysis showed a significant race × task × condition interaction: in non-Caucasians only, cathodal tDCS reduced accuracy in FP (sham = 73.4%, SD = 8%; c-tDCS = 63.7%, SD = 16%; *p* =.027) and CFMT (sham = 80.3%, SD = 13%; cathodal tDCS = 70.9%, SD = 15%; *p* =.046). No effect was observed in object-based tasks.
Payne and Tsakiris (2017)	60 (44 women), mean age of 21.54 years;	*Video-morphing Task* Behavioral measurements: Point of discrimination, Response timing.	No.	Application area: CP6; Intensity: 1 mA; Duration: 20 min. Electrode size: 35 cm²; Stimulation type: cathodal and anodal. tDCS type: offline. Sessions = 1 (between-subjects, pre-post design).	Anodal tDCS–induced modulation of excitability targeting the right temporoparietal area significantly affected self–other face discrimination. The percentage of self-face present at the point of discrimination increased from 50.46% pre-tDCS to 52.88% post-tDCS (*p* =.001).
Civile et al. (2018)	144 (111 women), mean age of 21 years;	*Old/New Recognition Task* Behavioral measurements: Accuracy, RTs.	No.	Application area: Fp3; Intensity: 1.5 mA; Duration: 10 min. Electrode size: 35 cm²; Stimulation type: anodal. tDCS type: online. Sessions = 1 (between-subjects, single-session design with study–test phases).	The reduction in the FIE caused by anodal tDCS applied to the Fp3 region was significant but not complete. In Experiment 1, the inversion effect in the Sham group had an effect size of ηp² = 0.62, whereas the anodal tDCS group showed a reduced effect size of ηp² = 0.34. Similarly, in Experiment 2, the Sham group had an effect size of ηp² = 0.71, and the anodal tDCS group showed a reduced effect size of ηp² = 0.27. This indicates that while the FIE was significantly reduced, it was not entirely eliminated.
Penton et al. (2018)	Experiment 1: 28 (17 women), mean age of 27.25 years. Experiment 2: 38 (17 women), mean age of 25.6 years. Experiment 3: 39 (30 women); younger adults: 19, mean age of 24.7 years; older adults: 20, mean age of 67.2 years.	CFMT, CCMT, Cambridge Bicycle Memory Test (CBMT). Behavioral measurements: Accuracy/percentage correct.	No.	Application area: F7/F8 (bilateral VLPFC); Intensity: 1.5 mA; Duration: 20 min. Electrode size: 25 cm²; Stimulation type: active high-frequency tRNS and sham. Type of non-invasive brain stimulation: high-frequency tRNS. Type of stimulation: partly offline and partly online. Experiment 1: Sessions = 1 (between-subjects, single-session pre-post design, same-day interval). Experiment 2: Sessions = 1 (between-subjects, single-session baseline-plus-post design, same-day interval). Experiment 3: Sessions = 2 (within-subjects, crossover design, 1-week interval).	Experiment 1: active tRNS improved face memory relative to sham, with a significant Stimulation Group × Session interaction, F (1,25) = 5.73, *p* =.024, η²*p* =.187. The active group improved from pre- to post-test, t (12) = 3.43, *p* =.005, whereas the sham group did not, t (13) = 0.759, *p* =.462; groups differed post-stimulation, t (25) = 2.09, *p* =.047. Experiment 2: tRNS did not modulate non-face visual memory. There was no main effect of Stimulation Group, F (1,36) = 0.02, *p* =.894, and no Task × Group interaction, F (1,36) = 0.31, *p* =.580. ANCOVA confirmed no effect on CCMT performance, F (1,35) = 0.17, *p* =.681. Experiment 3: active tRNS reduced face memory relative to sham, with a main effect of Stimulation Condition, F (1,37) = 5.267, *p* =.028, η²*p* =.125. Older adults performed worse overall than younger adults, F (1,37) = 9.226, *p* =.004, η²*p* =.200, but there was no Stimulation Condition × Age Group interaction, F (1,37) = 0.052, *p* =.820. Baseline performance predicted change after stimulation, β = −0.468, t = − 2.73, *p* =.010.
Gonzalez-Perez et al. (2019)	60 (30 women), mean age of 32.22 years.	Face Perception Task (FP), Object Perception Task (OP), CFMT, CCMT, CFPT (baseline only). Behavioral measurements: Accuracy and reaction time.	No.	Application area: PO8/FP1 and Cz/FP1; Intensity: 1.5 mA peak-to-peak; Duration: 20 min. Electrode size: 25 cm²; Stimulation type: sham, 5 Hz tACS, and 40 Hz tACS. Type of non-invasive brain stimulation: tACS. Type of stimulation: offline. Sessions = 1 (between-subjects, single-session design, no interval).	40 Hz occipital tACS enhanced perception performance relative to sham, with a main effect of condition, F (3,56) = 10.47, *p* <.001, ηp² = 0.37; mean accuracy was 77.9% in the 40 Hz_tACS group versus 68.0% in sham. No significant differences were found between sham and 5 Hz_tACS (*p* =.49) or between sham and Cz_40 Hz_tACS (*p* = 1.0), and 40 Hz_tACS outperformed Cz_40 Hz_tACS (*p* =.012). Memory performance was not modulated, F (3,56) = 1.62, *p* =.20.
Civile et al. (2019)	48 (39 women), mean age of 18.9 years;	*Old/New Recognition Task (with faces in vertical and inverted)* Behavioral measurements: Accuracy, RTs, Raw accuracy scores, Bias analysis.	No.	Application area: Fp3; Intensity: 1.2 mA; Duration: 10 min Electrode size: 25 cm²; Stimulation type: anodal tDCS type: online Sessions = 1 (mixed design, single-session with study–test phases)	The inversion effect in the anodal tDCS group was significantly smaller compared to the sham tDCS group (F (1, 46) = 7.45, *p* =.009, d = 0.78). This reduction highlights the impact of anodal tDCS on perceptual learning and face recognition performance.
Willis et al. (2019)	Experiment 1: 53 (43 women), mean age of 23.09 years; Experiment 2: 39 (26 women), mean age of 25,36 years;	*TOPF*,* CFMT*,* Emotion Hexagon*,* Facial Identity Task*,* Facial Expression Task*,* OP*, Behavioral measurements: Accuracy, RTs.	No.	Experiment 1: Application area: PO8/FP1; Intensity: 1.5 mA; Duration: 20 min. Electrode size: 25 cm²; Stimulation type: anodal tDCS and sham. Type of non-invasive brain stimulation: tDCS. Type of stimulation: offline. Sessions = 1 (between-subjects, single-session design, no interval). Experiment 2: Application area: PO7/PO8; Intensity: 1.5 mA peak-to-peak; Duration: 20 min. Electrode size: 25 cm²; Stimulation type: high-frequency tRNS and sham. Type of non-invasive brain stimulation: high-frequency tRNS (100–500 Hz). Type of stimulation: offline. Sessions = 1 (between-subjects, single-session design, no interval)	Neither anodal tDCS nor high-frequency tRNS significantly improved accuracy or reaction times for any task compared to sham (all ps > 0.14; ηp² ≤ 0.04). Bayes factors (BF10 < 0.52) supported the null hypothesis. Thus, both experiments failed to replicate previous findings of enhanced face perception with similar stimulation protocols and larger samples.
Alekseichuk (2020)	Experiment 1 (EEG): 20 (12 women). Experiment 2 (fMRI): 20 (12 women). Experiment 3 (tACS): 25 (13 women).	Face-value associative encoding task, Short-term recognition task, Long-term recognition task. Behavioral measurements: Accuracy, confidence, familiarity, and recollection.	EEG: theta power and Granger-causality/connectivity analyses. fMRI: generalized psychophysiological interaction (gPPI) connectivity analysis.	Application area: right posterior neocortex/parietal region (target: P4 with returns at T8, C2, CP1, Oz); active control: left posterior neocortex (P3 with returns at T7, C1, CP2, Oz). Frequency: 4 Hz; Intensity: 3 mA peak-to-peak; Duration: ~20 min. Electrode size: round rubber electrodes, radius = 1 cm; Stimulation type: target tACS, active control tACS, and sham. Type of non-invasive brain stimulation: tACS. Type of stimulation: online. Experiment 3: Sessions = 3 (crossover, ≥ 72 h interval).	EEG showed that right posterior theta during encoding predicted subsequent long-term recognition, F (1,1714) = 4.47, *p* =.035, whereas short-term performance was not affected, F (1,1702) = 2.58, *p* =.11. fMRI identified the right angular gyrus as the strongest encoding-related node, b = 1.21, F (12,8) = 29.84, pFDR = 0.003. In the tACS experiment, stimulation affected long-term but not short-term memory, F (2,13497) = 4.73, *p* =.009; F (2,6704) = 0.36, *p* =.70. Target stimulation improved overall long-term recognition versus sham, *p* =.023, Hedges’ g = 0.34, and increased familiarity versus sham, *p* =.032, and versus active control, *p* =.04, while recollection remained unchanged, *p* =.69.
Civile et al. (2020)	Experiment 1: 48 (30 women), mean age of 21.3 years; Experiment 2: 64 (49 women), mean age of 21.1 years;	*Old/New Recognition Task* Behavioral measurements: Accuracy, RTs, Sensitivity;	EEG: ERP components analyzed: *N170*	Application area: Fp3; Intensity: 1.5 mA; Duration: 10 min Electrode size: 35 cm²; Stimulation type: anodal tDCS type: online Sessions = 1 (between-subjects, pre-post recognition design)	Anodal tDCS reduced the behavioral face inversion effect and modulated the N170 ERP component. The latency-based inversion effect was reduced from 7.95 ms to 4.70 ms (Exp. 1a) and from 12.15 ms to 2.37 ms (Exp. 1b, study phase); during recognition (combined data), it decreased from 11.12 ms (sham) to 5.03 ms (tDCS). Additionally, the amplitude-based inversion effect increased from 0.96 µV (sham) to 1.81 µV (tDCS), F (1, 108) = 7.39, *p* =.008, ηp² = 0.06, indicating distinct modulation of configural face processing.
Civile et al. (2021a) ^a^	168 (120 women), mean age of 20,65 years.	*Face-matching task* (same and different stimuli for each stimulus type); *Old/New Recognition Task* Behavioral measurements: Accuracy, RTs, Sensitivity;	No.	Application area: Experiment 1: Fp3and Experiment 2: PO8; Intensity: 1.5 mA; Duration: 10 min Electrode size: 35 cm²; Stimulation type: anodal tDCS type: online Sessions = 1 (between-subjects)	Anodal tDCS over Fp3 significantly reduced overall performance in upright face recognition (Exp. 1a: 2.34 vs. 2.68, *p* =.030, ηp² = 0.10; Exp. 2: 1.94 vs. 2.44, *p* =.041) without affecting the composite face effect. The stimulation also reduced the face inversion effect, replicating prior findings. No effects were observed with active control stimulation over PO8, suggesting site specificity related to perceptual learning and face expertise.
Civile et al. (2021b) ^b^	96 (66 women), mean age of 20.9 years;	*Face-Matching Task*,* checkerboard-matching Task* Behavioral measurements: Hit rate, False Alarm Rate, Sensitivity Index.	No.	Application area: Fp3; Intensity: 1.5 mA; Duration: 10 min Electrode size: 35 cm²; Stimulation type: anodal tDCS type: online. Sessions = 1 (between-subjects)	Anodal tDCS over Fp3 significantly reduced the face inversion effect (sham = 0.822, anodal = 0.428; *p* =.006) but did not eliminate it. The checkerboard inversion effect was completely abolished (sham = 0.565, anodal = 0.002; *p* =.010).
Papagno et al. (2021)	Experiment 1: 32 patients with temporal lobe tumors (11 women), mean age of 41,75 years; Experiment 2: 64 healthy adults, assigned to 4 tDCS groups (*n* = 16 each), mean age of 23 years;	*UVD*,* VO-REC*,* FA-REC*,* Unknown Face Recognition Task* *and Benton Facial Recognition Test* Behavioral measurements: Familiarity judgment, semantic identification, and naming for faces and voices; false alarms (FA), sensitivity (d’), and response criterion (β) from signal detection theory.	No.	Application area : T3 e T4; Intensity: 1.5 mA; Duration: 20 min; Electrode size: anode 25 cm² e cathode 35 cm²; Stimulation type: anodal tDCS type: offline Sessions = 2 (mixed design, pre-post tDCS with between-subjects stimulation site and task modality factors, interval not specified)	Patients with right temporal lobe tumors showed more false alarms in voice (b = 0.23, *p* =.002) and face recognition (b = 0.13, *p* =.038) compared to those with left-sided tumors, along with lower sensitivity for voices (b = 0.72, *p* =.013) and a more lenient response criterion (β = 0.63, *p* =.019). No correlation was found between familiarity judgments and perceptual discrimination. In healthy participants, right ATL tDCS improved voice sensitivity (b = 0.37, *p* =.007) and marginally improved face recognition (b = 0.36, *p* =.053), whereas left ATL stimulation had no effect. These results support a stronger right-hemispheric contribution to nonverbal person recognition, particularly for voices.
Awasthi (2022)	25 (13 women), mean age of 26.36 years.	Sex categorization task with spatial-frequency filtered hybrid faces, presented in foveal and peripheral blocks. Behavioral measurements: Response time (accurate trials only).	No.	Application area: P8; return electrode: shoulder; Intensity: 1 mA; Duration: 20 min. Electrode size: 25 cm²; Stimulation type: high-frequency tRNS and sham. Type of non-invasive brain stimulation: high-frequency tRNS. Type of stimulation: online. Sessions = 2 (within-subjects, counterbalanced crossover, consecutive-day interval).	Active tRNS reduced response times relative to sham, F (1,24) = 8.05, *p* =.009, ηp² = 0.250. This effect depended on target condition, with a significant Stimulation × Target Condition interaction, F (1,24) = 4.47, *p* =.006, ηp² = 0.157, showing faster responses for incongruent targets in the no-distractor condition, t = 3.04, *p* =.006, and distractor-present condition, t = 3.75, *p* =.01, but not for congruent targets. Stimulation also interacted with location, F (1,24) = 9.35, *p* =.005, ηp² = 0.280, with faster responses at the periphery (mean difference = 39.46 ms, t = 4.1, *p* <.001), but not at the fovea, t = 0.43, *p* =.66.
Civile and McLaren (2022)	96 (62 women), mean age of 20.8 years;	*Old/New Recognition Task* Behavioral measurements: Sensitivity, Decision criterion.	No.	Application area: Fp3; Intensity: 1.5 mA; Duration: 10 min Electrode size: 35 cm²; Stimulation type: anodal tDCS type: online Sessions = 1 (mixed design, single-session design with study–test phases)	Anodal tDCS significantly reduced the FIE for own-race (Western Caucasian) faces compared to sham (t (94) = 3.02, *p* =.003, η² ≈ 0.08), contributing to the elimination of the own-race effect.
Kho et al. (2023a) ^a^	Participants: 90(67 women), mean age of 21.1 years;	*CFMT*,* CFMT-Chinese*,* CFMT-Chinese Malaysian and CFMT-Australian* Behavioral measurements: Accuracy, RTs	No.	Application area: PO8 e FP1; Intensity: 1.5 mA; Duration: 20 min Electrode size: 25 cm²; Stimulation type: cathodal and anodal tDCS type: offline Sessions = 2 (mixed design, pre-post design with between-subjects stimulation groups, same-day interval)	No significant differences were observed in the recognition of same-race and other-race faces following anodic, cathodic, or sham tDCS. These results suggest that tDCS may not effectively modulate performance in face recognition tasks.
Civile et al. (2023)	128 (88 women), mean age of 20.7 years;	*Old/new recognition phase*,* Categorization task* Behavioral measurements: Hit rate, False Alarm Rate.	No.	Application area: Fp3; Intensity: 1.5 mA; Duration: 10 min Electrode size: 25 cm²; Stimulation type: anodal tDCS type: online Sessions = 1 (mixed design, single-session design with study–test phases)	Anodal tDCS modulated the FIE depending on stimulus context. In Experiment 1a (normal + Thatcherized faces), FIE increased numerically in the tDCS group (M = 0.87) compared to sham (M = 0.67), but the difference was not significant. In Experiment 1b (normal faces + checkerboards), tDCS significantly reduced FIE (M = 0.29 vs. M = 1.10), indicating reduced configural disruption when no harmful generalization occurred.
Kho et al. (2023b) ^b^	60 (38 women), mean age 21.38 years;	*Facial Recognition Task* Behavioral measurements: Rate-Correct Score, Accuracy, Median reaction time for correct responses.	No.	Application area: FFA e OFA (right); Intensity: maximum current: 2 mA per electrode (4 mA total); Duration: 20 min Electrode size: 3.14 cm²; Stimulation type: anodal tDCS type: offline Sessions = 2 (mixed design, pre-post design with between-subjects stimulation groups and within-subjects CFMT type, same-day interval)	FFA stimulation improved facial feature recognition efficiency (M = 0.106, SD = 0.145) but had no effect on whole-face recognition (M = 0.013, SD = 0.125). OFA stimulation had no significant effect on either task.
Estudillo (2023)	52 (34 women), mean age of 21.25 years.	Type of tasks: Unfamiliar face matching task with high-resolution and pixelated faces. Behavioral measurements: Accuracy.	No.	Application area: P7/P8; Intensity: 1 mA; Duration: 20 min. Electrode size: 25 mm²; Stimulation type: high-frequency bilateral tRNS and sham. Type of non-invasive brain stimulation: high-frequency tRNS (100–500 Hz). Type of stimulation: offline. Sessions = 1 (between-subjects, single-session design, no interval).	Active high-frequency tRNS improved unfamiliar face matching accuracy relative to sham, F (1,50) = 10.16, *p* <.01, ηp² = 0.17, with mean accuracy of 75.75% in the active tRNS group versus 69.49% in the sham group. Accuracy was higher for high-resolution than pixelated faces, F (1,50) = 189.68, *p* <.001, ηp² = 0.79, and higher for mismatch than match trials, F (1,50) = 9.14, *p* <.01, ηp² = 0.15. No interactions were significant, all Fs (1,50) ≤ 1.38, all ps ≥ 0.20, indicating that the tRNS benefit was present for both unaltered and pixelated faces.
Civile et al. (2024)	Participants: 72(52 women), mean age of 20.4 years;	*Old/New Recognition Task* Behavioral measurements: Hit rate and False alarm rate.	EEG: ERP components analyzed: *N170*	Application area: Fp3; Intensity: 1.5 mA; Duration: 10 min Electrode size: 35 cm²; Stimulation type: anodal tDCS type: online Sessions = 1 (mixed design, single-session design with study–test phases)	Anodal tDCS over Fp3 increased the face inversion effect (t (35) = 5.94, *p* <.001, η²*p* =.50) compared to sham (t (35) = 2.96, *p* =.006, η²*p* =.20), driven by improved recognition of upright faces (M = 1.25 vs. M = 0.87; t (70) = 2.02, *p* =.047). tDCS also reduced N170 latency and revealed a dissociation between behavioral performance and amplitude (negative correlation).
Civile et al. (2025)	Experiment 1: 120 (86 women), mean age of 20.3 years; Experiment 2: 160 (112 women), mean age of 20.5 years; Experiment 3: 160 (120 women), mean age of 21.0 years. Total *N* = 440. All were University of Exeter students, randomly assigned to tDCS groups in a double-blind design.	Old/New Recognition Task with upright and inverted faces; study phase plus recognition phase.	No.	Application area: Fp3/Fp2; Intensity: 1.5 mA; Duration: 10 minutes per stimulation period Electrode size: 35 cm²; Stimulation type: sham, anodal, and cathodal; tDCS type: online; Double-blind. Sessions = 2 (between-subjects, study-phase and recognition-phase stimulation, same-day interval)	Experiment 1: Anodal–sham reduced the FIE compared with sham–sham, t (78) = 2.10, *p* =.038, and anodal–cathodal restored the effect relative to anodal–sham, t (78) = 2.64, *p* =.009. The effect was driven by poorer upright-face recognition in anodal–sham (M = 0.45) versus sham–sham (M = 0.66) and anodal–cathodal (M = 0.69). Experiment 2: Anodal–sham again reduced the FIE versus sham–sham, t (78) = 2.51, *p* =.014, whereas anodal–cathodal restored it, t (78) = 2.11, *p* =.037. Cathodal–sham did not differ from sham–sham, t (78) = 0.65, *p* =.51. The effect was again driven by lower upright-face recognition in anodal–sham (M = 0.40) compared with sham–sham (M = 0.62) and anodal–cathodal (M = 0.70). Experiment 3: Anodal–sham reduced the FIE relative to sham–sham, t (78) = 2.89, *p* =.024, and anodal–cathodal reversed this reduction, t (78) = 2.98, *p* =.024. Sham–cathodal did not differ from sham–sham, t (78) = 0.67, *p* =.50. Again, the effect was driven by reduced upright-face recognition in anodal–sham (M = 0.39) versus sham–sham (M = 0.63).
Saghravanian et al. (2025)	47 healthy participants, mean age of 27 years (SD = 6); sex/gender not specified. Randomly assigned to M1 (*n* = 13), M2 (*n* = 12), M3 (*n* = 13), and Sham (*n* = 9). Single-blind, sham-controlled.	Delayed Match-to-Sample face identification task with morphed faces.	No.	Application area: P8/P7; Intensity: 1.5 mA; Duration: 20 min. Electrode size: circular sponge electrodes, 5 cm diameter; Stimulation type: active and sham. Type of tDCS: offline. Sessions = 1 (between-subjects, single-session design, no interval)	M1 was the only montage that significantly improved face identification relative to sham, F (1,120) = 4.74, *p* =.031, with the strongest effect at the 30% morph level (66.53 ± 4.17% vs. 50.55 ± 3.76%, *p* <.01). M2, F (1,114) = 1.59, *p* =.209, and M3, F (1,120) = 2.46, *p* =.119, did not differ significantly from sham. RTs were longer in M1, F (1,120) = 4.67, *p* =.033, and M3, F (1,120) = 13.68, *p* =.0003.

Abbreviations: *CFPT* Cambridge Face Perception Test, *CFMT* Cambridge Face Memory Test, *CCMT* Cambridge Car Memory Test, *CBMT* Cambridge Bicycle Memory Test, *DLPFC* Dorsolateral Prefrontal Cortex, *FP* Face Perception task, *OFA* Occipital Face Area, *FFA* Fusiform Face Area, *FIE* Face Inversion Effect, *OP* Object Perception task, *ATL* Anterior Temporal Lobe, *tACS* Transcranial Alternating Current Stimulation, *UVD* Voice discrimination, *VO-REC* Voice recognition, *FA-REC* Face Recognition Task, *TOPF* Test of Premorbid Function, *LaRc* anode left/cathode right, *LcRa* cathode left/anode right, *tRNS* transcranial random noise stimulation, *RTs* Reaction Times, *EEG* Electroencephalography, *ERP* Event-Related Potentials, *tDCS* Transcranial Direct Current Stimulation

## Results

**Fig. 1:**
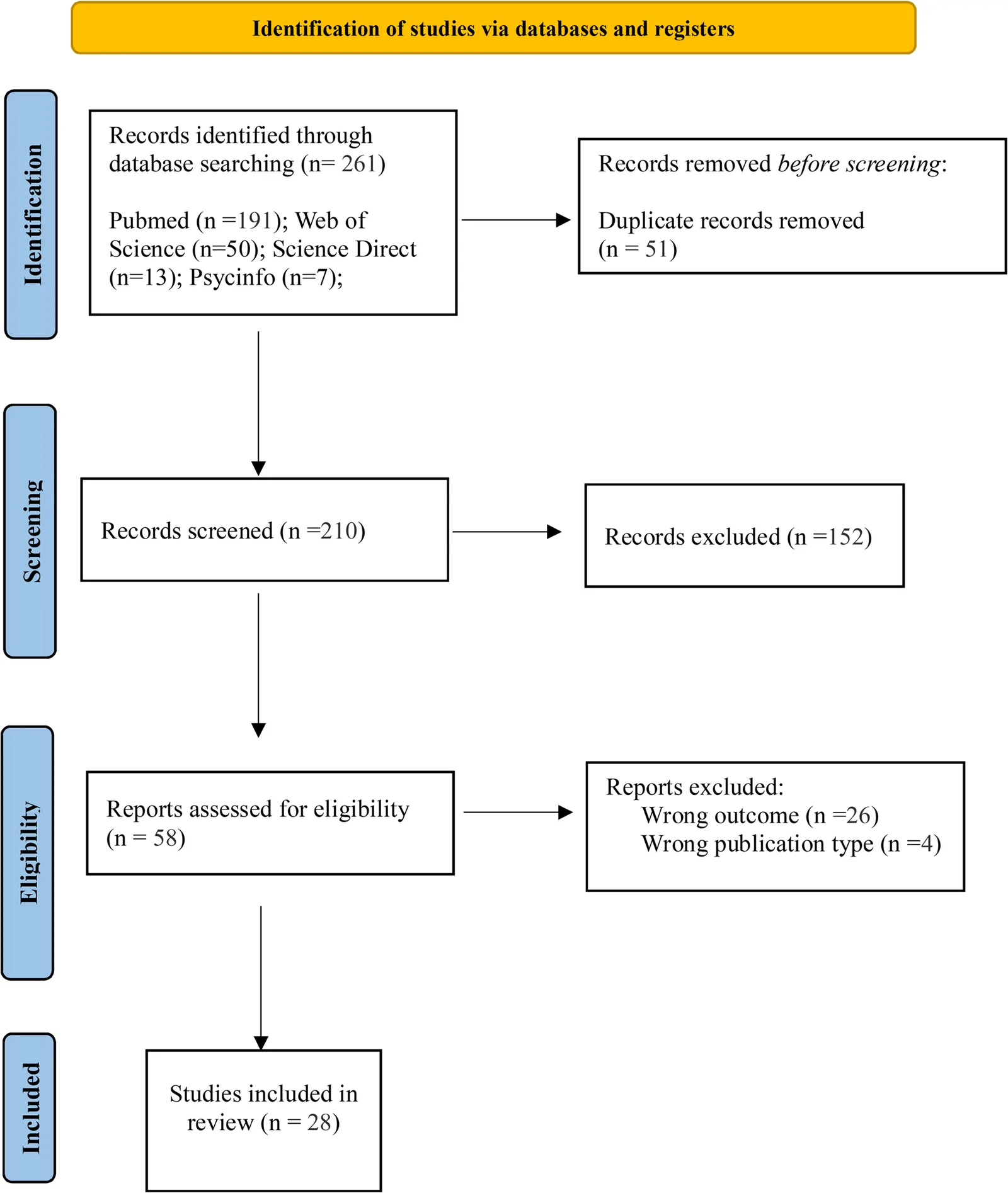
Flowchart of literature search and selection process

An initial search identified 261 articles. After screening titles and abstracts, 28 articles were included according to the eligibility criteria (Fig. 1). We found that 12 articles were published in the last five years, between 2021 and 2026, while the remaining studies were published between 2014 and 2020. The total sample size across all included studies was 2,064 participants, varying from 32 (Papagno et al., 2021; Renzi et al., 2015) to 440 participants per study (Civile et al., 2025), with most of the sample consisting of women (*n* = 1,388; approximately 67.25%).

Among the 28 included studies, only one included a sample composed of clinical patients with unilateral anterior temporal lobe resection (Papagno et al., 2021). The remaining studies were conducted with healthy volunteers. Regarding the type of blinding, 12 studies employed double-blinding, 10 used single-blinding, and 6 did not provide information about the blinding procedure.

As for the tDCS protocol used, the stimulation duration ranged from 10 to 24.6 min, with an intensity of 0.5 to 4 mA; 1.5 mA was the most commonly used intensity (*n* = 22). Electrode sizes ranged from 3.14 cm² to 35 cm², with the 35 cm² electrodes being the most used (*n* = 15), and anodal stimulation was the most frequently chosen type. The most frequently targeted stimulation area was the left dorsolateral prefrontal cortex (Fp3 according to the EEG 10–20 system; *n* = 14), followed by the right occipitotemporal cortex (PO8; *n* = 7). Online tDCS (i.e., effects measured during stimulation) was slightly more common (*n* = 14), followed by offline tDCS (*n* = 11), with three studies adopting a combined online/offline design (Barbieri et al., 2016; Yang et al., 2014; Penton et al., 2018).

Regarding other non-invasive brain stimulation techniques, tRNS and tACS were also employed in a subset of studies. For tRNS (*n* = 5), high-frequency stimulation (100–500 Hz) was the most common protocol, with intensity of 1–1.5 mA, duration of 20 min, and electrode size of 25 cm². The primary target areas were bilateral occipitotemporal cortices (P7/P8) and bilateral ventrolateral prefrontal cortex (F7/F8). tRNS was applied either offline (Romanska et al., 2015; Willis et al., 2019; Estudillo, 2023) or in a combined online/offline design (Penton et al., 2018). Results showed that tRNS improved face perception and memory in some studies (Romanska et al., 2015; Penton et al., 2018; Estudillo, 2023), although null findings were also reported (Willis et al., 2019). Regarding tACS (*n* = 2), one study applied 40 Hz occipital tACS (PO8/FP1) and found enhanced face perception compared to sham (Gonzalez-Perez et al., 2019), while another employed 4 Hz theta tACS over the right posterior parietal region and observed improved long-term face recognition memory (Alekseichuk et al., 2020). These findings suggest that both tRNS and tACS can modulate face-related cognitive processes, though the number of studies remains limited. Regarding the number of stimulation sessions across all 28 studies, 16 employed a single session, while 12 used two or three sessions (crossover or pre-post designs). No study applied repeated stimulation over multiple weeks.

Studies employed tDCS combined with various neuropsychological instruments such as the Cambridge Face Memory Test (Barbieri et al., 2016; Costantino et al., 2017; Kho et al., 2023^b^; Willis et al., 2019), Benton Recognition Test (Papagno et al., 2021), Orientation Judgment Task (Yang et al., 2014), and Old/New Recognition Task (Civile et al., 2021^a^; Civile et al., 2016; Civile et al., 2018, 2020, 2023; Civile et al., 2021^b^; Civile et al., 2025). Additionally, tRNS studies employed the CFMT (Penton et al., 2018; Willis et al., 2019), CFPT (Romanska et al., 2015), and face matching tasks (Estudillo, 2023), while tACS studies used the CFMT and CFPT (Gonzalez-Perez et al., 2019) as well as face-value associative encoding tasks (Alekseichuk et al., 2020).

Electrophysiological findings demonstrate that tDCS directly modulates the N170 component. Yang et al. (2014) found that tDCS over P7/P8 reduced right-hemisphere N170 amplitude, correlating with decreased holistic face processing. Civile et al. (2020, 2024) extended this by showing that anodal tDCS over Fp3 alters both N170 amplitude and latency in face inversion paradigms, with the latter study revealing a dissociation between ERP measures and behavioral performance. Additionally, Alekseichuk et al. (2020) used EEG to show that tACS over right posterior cortex enhances theta power during encoding, which predicts long-term face recognition. Together, these findings confirm that non-invasive stimulation systematically alters early, face-selective neural activity.

Most of the studies (21 out of 28) demonstrated moderate methodological quality based on the PEDro scale, indicating that while these studies met some main validity criteria, they likely had methodological limitations, such as a lack of adequate blinding, absence of concealed allocation, or failures in intention-to-treat analysis. These factors may introduce some level of bias in the results. However, the presence of seven high-quality studies (scoring between 8 and 10 on the PEDro scale) strengthens the conclusions of this systematic review, offering more robust and reliable evidence.

## Discussion

This systematic review compiled evidence from 28 studies examining the effects of TES on facial identity identification, as well as on related perceptual processes (e.g., holistic processing, face inversion effect) that support identification. Given the heterogeneity in outcomes assessed, the discussion is organized thematically, with distinct sections dedicated to different outcome domains and their corresponding stimulation targets. Critically, throughout this discussion, we distinguish between studies that directly measured face identification (e.g., CFPT, CFMT, old/new recognition tasks) and those that measured face perception without explicit identification demands (e.g., orientation judgment, Mooney faces, composite face tasks). This distinction is essential for interpreting the functional specificity of non-invasive brain stimulation effects.

Before examining specific findings, it is necessary to clarify how we interpret stimulation induced changes in face processing. In this review, enhancement refers to any stimulation effect that improves behavioral performance (accuracy, response time, or memory) relative to sham, or that increases a typical marker face processing (e.g., a larger face inversion effect driven by improved upright recognition). Conversely, disruption refers to any stimulation effect that impairs performance or reduces a canonical marker (e.g., a smaller face inversion effect due to impaired upright recognition). We also clarify that some outcomes, such as reduced face inversion effect, are not univocal in their interpretation. Depending on the underlying mechanism, such changes may reflect either a loss of configural processing (disruption) or a shift toward processing strategies (which may not necessarily be detrimental). Thus, throughout this discussion, we explicitly state whether an effect is interpreted as enhancement or disruption based on the task demands and the direction of change relative to typical behavior in healthy adults. This framework avoids the ambiguity of describing effects simply as “modulation” without functional specification.

### Effects of tDCS on the face inversion effect

The FIE is widely considered a perceptual phenomenon reflecting the disruption of configural and holistic processing mechanisms (Rossion, 2008). Although the FIE has implications for face identification, it primarily indexes perceptual stages of face processing, as tasks measuring this effect typically do not require explicit identity recognition. Accordingly, the studies reviewed in this section are better interpreted as reflecting the modulation of face perception, rather than identity processing.

A central question emerging from the literature is how to interpret a reduction in the FIE following tDCS. The Civile group has consistently showed that anodal tDCS over Fp3 disrupts the perceptual expertise that normally facilitates upright face recognition (Civile et al. 2016, 2018, 2019, 2020, 2021b). This interpretation is supported by the finding that stimulation selectively impairs performance for upright faces, rather than improving performance for inverted ones, an effect we interpret as disruption of configural expertise. However, an alternative account is that reduced FIE reflects a shift toward more featural or analytic processing strategies, which may be adaptive in certain contexts. More recent findings, however, challenge the notion that anodal tDCS uniformly disrupts configural processing: Civile et al. (2024) reported that anodal tDCS over Fp3 increased the behavioral FIE, driven by improved recognition of upright faces, an effect we interpret as enhancement of configural processing. This suggests that the functional outcome depends on contextual factors, such as task parameters, stimulation montage, or sample characteristics.

Further complexity emerges from studies combining tDCS with EEG. Civile et al. (2020) demonstrated that anodal tDCS over Fp3 produced opposite effects on different measures of the N170 component: the latency-based inversion effect was reduced (interpreted as disruption of processing speed), whereas the amplitude-based inversion effect was increased (interpreted as enhancement of neural resource allocation). This dissociation within the same participants suggests that tDCS does not exert a unitary influence on face processing, but rather modulates multiple partially independent neural mechanisms. Critically, these neural changes did not map directly onto behavioral performance, indicating a complex and non-linear relationship.

The effects of tDCS on the FIE also depend critically on timing and context. Civile et al. (2025) showed that the disruptive effect of anodal tDCS during encoding could be reversed by subsequent cathodal stimulation during retrieval, indicating that tDCS effects are not fixed but dynamically modulated. Civile et al. (2023) further demonstrated that the same stimulation protocol can produce opposite effects depending on stimulus context: when upright faces were presented alongside Thatcherized faces, tDCS increased the FIE; when presented alongside checkerboards, tDCS reduced the FIE. Additionally, Civile et al. (2021b) found that anodal tDCS eliminated the inversion effect for checkerboards (a non-face stimulus), but only reduced it for faces, suggesting that the FIE may rely on both an expertise-based component (sensitive to tDCS) and a face-specific component (relatively resistant to tDCS).

Site specificity has also been demonstrated: Civile et al. (2021a b a) showed that anodal tDCS over Fp3 reduced the FIE, whereas stimulation over PO8 had no effect, supporting a top-down modulatory role of the DLPFC. Finally, extending beyond standard laboratory stimuli, Civile and McLaren (2022) demonstrated that tDCS over Fp3 eliminated the own-race advantage, interpreted as disruption of race-specific perceptual expertise, suggesting that the perceptual expertise disrupted by tDCS generalizes to socially relevant face categories.

In summary, anodal tDCS over the left DLPFC reliably modulates the FIE, but the direction of this modulation (reduction/disruption or enhancement) depends on contextual and methodological factors. Overall, the literature supports the interpretation that changes in the FIE primarily reflect modulation of perceptual (configural/holistic) processing, with FIE reduction generally interpreted as disruption of expertise rather than enhancement. Importantly, because all studies reviewed here were conducted in healthy young adults, the clinical relevance of these findings remains speculative and requires dedicated investigation in patient samples.

### Effects of tDCS on the composite face effect

The composite face effect (CFE) is a behavioral marker of holistic face processing, referring to the difficulty of perceiving the top half of a face as independent from the bottom half when the two halves are aligned to form a typical face, as opposed to when they are misaligned. A reduction in the composite effect is typically interpreted as a decrease in holistic integration, indicating that the two halves are being processed more independently. Like the face inversion effect, the composite face effect indexes perceptual mechanisms that support, but are not equivalent to, explicit face identification. Accordingly, modulation of this effect reflects changes in how faces are perceptually integrated, rather than changes in identity recognition per se.

Only three studies have directly examined the effects of tDCS on the composite face effect, and their findings are notably divergent. Yang et al. (2014) applied tDCS over posterior temporal regions (P7/P8) and found a significant reduction in the composite effect, interpreted as disruption of holistic processing. This effect was accompanied by reduced N170 amplitude in the right hemisphere, providing convergent electrophysiological evidence for the modulation of configural face processing.

In contrast, two other studies reported null effects. Renzi et al. (2015) applied anodal tDCS over the OFA and found no significant modulation of the composite face effect, despite observing impairments in face and object detection under degraded conditions using Mooney faces. The authors concluded that OFA is not directly implicated in holistic processing involved in face discrimination, although it plays a causal role in facial detection when the signal is degraded. Similarly, Civile et al. (2021a) examined the composite face effect following anodal tDCS over Fp3 and over PO8 and found no significant modulation for either stimulation site. Critically, in the same study, tDCS over Fp3 reliably reduced the face inversion effect, demonstrating that the null effect on the composite task was not due to ineffective stimulation.

This dissociation between the composite face effect and the face inversion effect is theoretically important. Although both phenomena are often considered markers of holistic processing, they may reflect partially distinct mechanisms. The composite face effect emphasizes the mandatory integration of face halves, whereas the face inversion effect is more closely tied to configural processing of spatial relations among facial features. The finding that tDCS over Fp3 reduces the inversion effect but not the composite effect suggests that prefrontal stimulation may selectively target configural processing mechanisms without disrupting the mandatory holistic integration indexed by the composite task. Conversely, stimulation over posterior temporal regions (P7/P8) reduced the composite effect (Yang et al., 2014), whereas stimulation over the OFA (Renzi et al., 2015) and over PO8 (Civile et al. 2021a) did not. This pattern suggests that different occipitotemporal subregions may play distinct roles in holistic integration.

Several factors may account for the observed variability across studies, including differences in stimulation parameters (current intensity, duration, electrode size), task versions, and sample characteristics. However, the most informative conclusion from this small body of literature is that the composite face effect and the face inversion effect are not interchangeable. They likely rely on partially non-overlapping neural substrates and are differentially sensitive to tDCS. From a functional perspective, reductions in the composite face effect, when observed, are interpreted as disruption of holistic processing. However, given the limited number of studies and the absence of independent replications for some findings, conclusions regarding the modulation of the composite face effect by tDCS remain preliminary.

### Effects of tDCS on perceptual learning paradigms

Perceptual learning refers to the improvement in perceptual discrimination ability resulting from repeated exposure to stimuli, often without explicit feedback or conscious awareness of what is being learned. In the context of face processing, perceptual learning paradigms typically involve repeated presentation of faces, and the outcome of interest is how prior exposure modifies subsequent performance. Unlike the face inversion effect or composite face effect, which measure online perceptual processing, perceptual learning paradigms assess changes in perceptual representations over time as a function of experience. The studies discussed in this section therefore examine whether tDCS can interfere with, enhance, or reverse the learning of face-related perceptual information.

A series of studies by Civile and colleagues has systematically investigated the effects of anodal tDCS over Fp3 on perceptual learning for faces. Civile et al. (2016) demonstrated that anodal tDCS during a study phase not only eliminated but also reversed perceptual learning, as indexed by the face inversion effect. This finding was interpreted as evidence that tDCS over Fp3 interferes with the formation of perceptual expertise for faces, an effect we interpret as disruption of expertise formation. Subsequent studies replicated and extended this finding, showing that anodal tDCS over Fp3 consistently disrupts perceptual learning, as reflected in a reduced or eliminated face inversion effect (Civile et al. 2018, 2019, 2020, 2021b). Civile et al. (2020) further showed that this disruption is accompanied by opposite effects on different measures of the N170 component (reduced latency-based inversion effect but increased amplitude-based inversion effect), indicating that tDCS affects multiple mechanisms within the perceptual learning process.

The effects of tDCS on perceptual learning are not uniform across stimulus categories. Civile et al. (2021b) found that anodal tDCS over Fp3 completely abolished the inversion effect for checkerboards (a non-face stimulus with no pre-existing expertise) but only reduced it for faces. This suggests that perceptual learning for faces may be supported by both an expertise-based component (sensitive to tDCS) and a face-specific component (relatively resistant to tDCS). Similarly, Civile and McLaren (2022) demonstrated that tDCS over Fp3 eliminated the own-race advantage, interpreted as disruption of race-specific perceptual expertise, indicating that the perceptual expertise disrupted by tDCS extends to socially relevant face categories. Civile et al. (2023) further showed that the effects of tDCS on perceptual learning are context-dependent: when normal faces were presented alongside Thatcherized faces, tDCS increased the inversion effect (though not significantly), whereas when presented alongside checkerboards, tDCS reduced it. This context dependency suggests that perceptual learning effects are not fixed but can be modulated by the presence of competing stimulus categories.

Civile et al. (2024) reported an intriguing reversal of the typical pattern: anodal tDCS over Fp3 increased the behavioral face inversion effect, driven by improved recognition of upright faces, an effect we interpret as enhancement of perceptual learning. More recently, Civile et al. (2025) introduced a dual-phase stimulation design and found that the disruptive effect of anodal tDCS during encoding could be reversed by subsequent cathodal stimulation during retrieval, indicating that the effects of tDCS on perceptual learning are not permanent but dynamically modulable.

Beyond the Civile group’s studies, Awasthi (2022) examined perceptual learning using a different approach: a sex categorization task with spatial-frequency filtered hybrid faces presented in foveal and peripheral blocks. High-frequency tRNS over P8 reduced response times relative to sham, particularly for incongruent targets and for stimuli presented in the periphery. The faster responses for peripheral stimuli (mean difference of approximately 40 ms) suggest that tRNS facilitated perceptual processing for stimuli presented outside the fovea, where face perception is typically more challenging. Functionally, this effect is interpreted as enhancement of perceptual processing speed, in contrast to the disruption account favored by the Civile group. However, Awasthi (2022) measured response times rather than accuracy or learning over time, and used tRNS rather than tDCS, making direct comparisons with the perceptual learning studies described above difficult.

In summary, the literature on tDCS and perceptual learning for faces is dominated by studies from a single research group. The majority of these studies report that anodal tDCS over Fp3 disrupts perceptual learning, as indexed by a reduction or elimination of the face inversion effect. This disruption is interpreted as interference with the formation or expression of configural or expertise-based mechanisms. However, under specific conditions (i.e., when normal faces are presented alongside Thatcherized faces), the same tDCS procedure can instead enhance the inversion effect (Civile et al., 2023, 2024), indicating that the effect of tDCS is context-dependent rather than uniformly disruptive. Additionally, one study using tRNS reported facilitatory effects on perceptual processing speed for peripheral stimuli (Awasthi, 2022).

### Effects of Non-invasive brain stimulation on face identification assessed by standardized instruments

Face identification refers to the ability to discriminate or recognize individual faces based on their unique features. Unlike the perceptual phenomena discussed in previous sections, face identification tasks require explicit judgments about whether a face is the same as another or whether it has been encountered before. The studies in this section have used a variety of standardized instruments, including the CFMT, the CFPT, and old/new recognition tasks. A critical distinction within this literature is that some tasks involve long-term memory (e.g., CFMT, old/new recognition), whereas others are simultaneous matching tasks (e.g., CFPT). These different task demands likely engage partially distinct neural mechanisms, and tDCS effects may not generalize across them.

### Facilitatory effects

A number of studies have reported facilitatory effects of non-invasive brain stimulation on face identification. Romanska et al. (2015) applied high-frequency tRNS over lateral occipitotemporal cortices (P7/P8) and found selective improvement on the CFPT-Identity for upright faces, with no effect on inverted faces or on trustworthiness judgments, an effect interpreted as enhancement of perceptual identification of facial identity without affecting higher-order social judgments. Similarly, Barbieri et al. (2016) showed that anodal tDCS over the right occipital cortex (PO8) delivered offline improved accuracy on the CFMT by approximately 7%, an effect that also generalized to object memory (CCMT), indicating that occipital stimulation may enhance visual perception broadly rather than specifically for faces. Brunyé et al. (2017) targeted the PO10 and found that anodal tDCS improved working memory for faces under high memory loads (3 and 4 items), with individuals showing lower baseline performance benefiting the most.

Using a different technique, Gonzalez-Perez et al. (2019) applied 40 Hz occipital tACS (PO8/FP1) and found enhanced perception of faces and objects, but no modulation of memory performance on the CFMT or CCMT. This dissociation suggests that tACS at gamma frequency may affect early perceptual stages without influencing later memory consolidation or retrieval. Penton et al. (2018) used high-frequency tRNS over bilateral ventrolateral prefrontal cortex (F7/F8) and reported improved CFMT performance in younger adults following stimulation, but a detrimental effect in older adults, highlighting the importance of age as a modulating factor. Baseline performance predicted the magnitude of tRNS effects, with lower-performing individuals showing greater benefits.

Studies using more complex or naturalistic stimuli have also contributed to the literature. Alekseichuk et al. (2020) applied 4 Hz tACS over the right posterior parietal cortex (P4) and found that stimulation enhanced long-term recognition memory for face-value associations, specifically increasing familiarity-based recognition without affecting recollection. EEG and fMRI data confirmed that right posterior theta activity during encoding predicted subsequent long-term memory, providing convergent neural evidence. Papagno et al. (2021) tested patients with unilateral temporal lobe tumors and healthy participants receiving anodal tDCS over the left or right anterior temporal lobe (ATL). In healthy participants, right ATL stimulation improved voice sensitivity and marginally improved face recognition, supporting a right-hemispheric contribution to nonverbal person recognition. Estudillo et al. (2023) applied high-frequency tRNS over bilateral occipitotemporal cortex (P7/P8) and found improved unfamiliar face matching accuracy for both high-resolution and pixelated faces, indicating that tRNS benefits persist even under degraded viewing conditions.

### Null and disruptive effects

However, the literature also contains notable null findings and failures to replicate. Willis et al. (2019) conducted two experiments with larger samples than previous studies and found no significant effects of anodal tDCS or high-frequency tRNS over occipitotemporal cortex on any face perception or identification task. Similarly, Kho et al. (2023 b) reported no effects of anodal or cathodal tDCS on own-race or other-race face recognition, indicating that tDCS over occipitotemporal regions may not reliably modulate face memory performance. Kho et al. (2023 a) used multifocal tDCS to target the fusiform face area (FFA) and OFA and found that FFA stimulation improved facial feature recognition efficiency but had no effect on whole-face recognition, whereas OFA stimulation produced no significant effects. This suggests that different subregions within the face-selective network may contribute differentially to feature-based versus holistic identification processes.

Beyond CFMT and CFPT, other identification tasks have yielded mixed results. Smirni et al. (2015) applied cathodal tDCS over the right DLPFC (F4) and found improved nonverbal recognition memory accuracy, suggesting that inhibition of right prefrontal regions may paradoxically enhance memory for faces. Payne and Tsakiris (2017) targeted the right temporoparietal area (CP6) and found that anodal tDCS increased the amount of self-face information required for discrimination, effectively inhibiting self-recognition, an effect interpreted as disruption of self-face processing. This finding is functionally opposite to enhancement, indicating that tDCS can impair identification depending on the task and target. Costantino et al. (2017) reported no overall effect of cathodal tDCS over PO8 on face or object tasks, but an exploratory analysis revealed a race × task × condition interaction: in non-Caucasian participants, cathodal tDCS reduced accuracy on the CFPT and CFMT, suggesting that stimulation effects may interact with participant characteristics such as race and baseline expertise.

Finally, a highly unusual finding comes from Saghravanian and Esteky (2025), who tested different tDCS montages on a delayed match-to-sample task with morphed faces. Surprisingly, only stimulation over the primary motor cortex (M1) improved face identification, particularly at the 30% morph level, whereas occipitotemporal montages (P8/P7) produced no significant effects. This result challenges the assumption that face identification is best modulated by stimulating face-selective regions and raises important questions about the specificity of tDCS effects.

## Overall methodological limitations and future directions

Across the 28 studies reviewed, several recurring methodological limitations compromise the comparability, replicability, and generalizability of the findings. First, although 16 studies employed a single session and 12 studies used two or three sessions (crossover or pre-post designs), none of the studies applied repeated stimulation over multiple weeks or months. This is an important limitation because the effects of non-invasive brain stimulation on face processing have only been examined in acute, transient paradigms. Repeated stimulation protocols are known to induce more persistent neuroplastic changes in other domains, such as motor learning and working memory, but such protocols have not yet been tested for facial identity recognition or face perception. Consequently, the generalizability of these findings to clinical or rehabilitation settings, where multiple sessions would be required, remains unknown.

Second, a substantial proportion of the included studies (approximately 60%, corresponding to 17 of 28 studies) were conducted by the same research group (Civile and colleagues). This concentration introduces potential bias. It leads to conceptual clustering, where similar theoretical interpretations (e.g., “disruption of perceptual expertise”) are consistently applied, potentially reinforcing a single explanatory framework without adequate testing of alternative accounts. It also results in methodological similarity, as most of these studies used the same montage (anodal tDCS over Fp3), similar tasks (inversion effect, perceptual learning), and comparable samples (healthy young adults), limiting generalizability to other montages, tasks, and populations. Moreover, independent replications from other laboratories are scarce; studies that attempted replication (e.g., Willis et al., 2019; Kho et al., 2023 a, 2023 b) often reported null or divergent findings. While the Civile group’s contributions are invaluable for proof-of-concept, the field would benefit from greater involvement of independent research groups using diverse methodologies, and we urge cautious interpretation of the apparent consistency in the literature.

Third, regarding clinical translation, all 28 studies were conducted with healthy young adults, typically university students. Although several studies mention potential clinical implications for prosopagnosia or other face recognition deficits, the present findings cannot be directly translated to clinical populations. Neural mechanisms in healthy individuals likely differ from those in patients with structural lesions or atypical networks, and stimulation protocols optimized in healthy adults may produce different or even detrimental effects in clinical populations. Furthermore, the reported effects are not consistently facilitatory; in many cases, particularly with anodal tDCS over Fp3, stimulation disrupts expertise-related effects, which would be undesirable in populations already suffering from face recognition deficits. Additionally, as noted, none of the studies employed repeated stimulation over multiple weeks or months, whereas clinical rehabilitation would require such prolonged protocols. A recent systematic review by Gobbo et al. (2023) concluded that effective protocols for prosopagnosia rehabilitation remain lacking. Therefore, we strongly caution against premature translation; any clinical application remains speculative until tested in dedicated patient samples with repeated-session designs and clinically relevant outcome measures.

Fourth, additional methodological limitations are evident. Sample sizes were often small, with several studies including fewer than 20 participants per experimental group (e.g., Barbieri et al., 2016; Renzi et al., 2015; Smirni et al., 2015), reducing statistical power and increasing the risk of both Type I and Type II errors. Although several studies adopted double-blind sham-controlled designs, others failed to clearly specify blinding procedures or did not include active control conditions, limiting causal interpretation. Most studies used large sponge electrodes (typically 5 × 7 cm), resulting in broad current spread and limited precision in targeting specific regions such as the fusiform face area or occipital face area. Only a few studies adopted more advanced techniques such as multifocal tDCS (Kho et al., 2023 a) or high-definition tDCS (Brunyé et al., 2017), which offer improved focality. Anatomical precision was generally limited by reliance on the 10–20 EEG system without individual neuroimaging; a notable exception is Renzi et al. (2015), who used stereotactic navigation based on individual MRI data to target the occipital face area. The predominance of healthy young adult samples limits external validity and generalization to other age groups and clinical populations, and only a few studies reported conducting neuropsychological screening prior to stimulation to ensure that participants’ cognitive performance was within the normative range.

To address these limitations, future research should: increase sample sizes and use pre-registered experimental designs; systematically compare single versus repeated stimulation protocols, including follow-up assessments to evaluate durability of effects; adopt more focal stimulation techniques (e.g., HD-tDCS, multifocal tDCS) and individualized target localization using structural or functional neuroimaging; include more diverse participant populations, including older adults and individuals with clinical conditions affecting face recognition (e.g., prosopagnosia, stroke); and conduct independent replications of key findings. By addressing these limitations, future studies will be better positioned to produce more robust, replicable, and translatable results, ultimately advancing neuromodulation protocols for cognitive research and clinical applications.

## Data Availability

No datasets were generated or analysed during the current study.
